# Effects and mechanisms of adipose tissue–derived extracellular vesicles in vascular inflammation and dysfunction

**DOI:** 10.4103/NRR.NRR-D-24-01619

**Published:** 2025-03-25

**Authors:** Daphne Lintsen, Bieke Broux

**Affiliations:** University MS Center, Campus Diepenbeek, Diepenbeek, Belgium; Neuro-Immune Connections and Repair Lab, Department of Immunology and Infection, Biomedical Research Institute, Hasselt University, Diepenbeek, Belgium

Neuroinflammation is a key process in the pathogenesis of various neurodegenerative diseases, such as multiple sclerosis (MS), Alzheimer’s disease, and traumatic brain injury. Even for disorders historically unrelated to neuroinflammation, such as Alzheimer’s disease, it is now shown to precede pathological protein aggregations. A common factor in these neurodegenerative diseases is activation of brain resident immune cells, as well as breakdown of the blood–brain barrier (BBB), which consecutively causes infiltration of peripheral immune cells, leading to neuronal damage and dysfunction. The integrity of the BBB, a biological barrier between the blood and the central nervous system, therefore plays a crucial role in regulating neuroinflammation. The BBB primarily consists of endothelial cells (ECs) that are tightly connected by tight junction proteins, astrocyte-end feet, and pericytes, conjunctively restricting the infiltration of immune cells and unnecessary biological molecules into the brain. BBB disruption, marked by the loss of structural integrity and upregulation of adhesion molecules and chemokines on the microvascular ECs, leads to infiltrating immune cells in the brain, driving neuroinflammation (Naegele and Martin, 2014).

Obesity, a growing global health concern, is a well-known risk factor for endothelial dysfunction and vascular impairments. Moreover, early childhood and adolescent obesity is a significant risk factor for the development of MS and other neurodegenerative disorders. Obesity is a chronic metabolic disease characterized by the accumulation of adipose tissue (AT). Recently, it has been acknowledged for its harmful impact on the central nervous system and the BBB. More specifically, obesity has been associated with dysregulated transport across the BBB and its breakdown. For instance, obesity causes the infiltration of monocytes and T-cells into the central nervous system by an increase in BBB permeability (Davanzo et al., 2023).

Research has shown that AT-derived extracellular vesicles (ATDEVs) contribute to the development of AT dysfunction, which has been associated with the initiation and disease progression of several obesity-related comorbidities including cardiovascular disease (CVD) (Delgadillo-Velázquez et al., 2022). Extracellular vesicles (EVs) are membranous nano-sized particles derived from cells and have emerged as pivotal inter-organ communication mediators, exerting diverse cellular processes depending on their molecular cargo. Once considered cellular waste, EVs are now understood to play an active role in transporting a range of complex biomolecules—proteins, lipids, and nucleic acids—that can influence target cell function and behavior. As carriers of non-secreted molecules, EVs enable the protected transfer of their contents across biological barriers, where they mediate diverse cellular processes. From modulating immune responses and tissue repair to influencing nervous system communication, EVs are fundamental in maintaining cellular homeostasis and coordinating responses to disease and injury. ATDEVs are generally secreted by adipocytes and other adipose tissue cells, and facilitate inter-organ communication, thereby playing a key role in metabolic regulation. In physiological states, ATDEVs contribute to metabolic homeostasis, yet under conditions like obesity and type 2 diabetes, they can drive pathological changes that exacerbate metabolic dysfunction and inflammation. Notably, ATDEVs’ lipid cargo plays a significant role in modulating immune responses, promoting a cycle of inflammation within AT through interactions with immune cells such as macrophages (Connolly et al., 2021). Current evidence shows an increase in ATDEV secretion in obese patients, thereby emphasizing their role in obesity-related disorders and highlighting the need for a better understanding of the role of ATDEVs in AT biology and its implications. In this perspective, we highlight some key mechanisms through which ATDEVs affect AT functioning and vascular integrity by focusing on vascular inflammation. We propose that these interactions are of particular interest to neurodegenerative impairments, as we hypothesize that ATDEVs could contribute to neurovascular impairment by altering BBB integrity and, by that, promote neuroinflammation.

**Obesity, adipose tissue dysfunction, and adipose tissue–derived extracellular vesicle secretion:** The AT is a metabolic organ facilitating energy homeostasis by regulation of systemic glucose and lipid metabolism through the secretion of active biomolecules, so-called adipokines. Dysfunction of the AT in obesity, including hypoxia and inflammation, leads to an increased risk of cardiometabolic diseases. Recently, several papers have described the critical role of ATDEVs in AT dysfunction. For instance, endoplasmic reticulum stress was shown to be triggered by ATDEVs (Fang et al., 2022). Stress in the endoplasmic reticulum, a key organelle in protein folding, lipid synthesis, and calcium storage, is a critical factor that exacerbates the pathophysiology of metabolic disorders such as insulin resistance, obesity, and cardiovascular disease. Endoplasmic reticulum stress is characterized by the accumulation of unfolded or misfolded proteins which triggers the unfolded protein response. This will eventually lead to an inflammatory cascade in the AT through the unfolded protein response, as well as a significant increase in the production of reactive oxygen species (Connolly et al., 2021). In another example, secretion of adipocyte-derived EVs is increased in hypoxic conditions, which is common in obese AT. As a consequence, these hypoxia-induced adipocyte-derived EVs impair insulin action in neighboring adipocytes through downregulation of the AKT pathway, thereby contributing to impaired metabolism and increased co-morbidities (Mleczko et al., 2018). Finally, AT-resident macrophages are the most abundant immune cells in obese AT (45%–50% of the stromal vascular fraction), and their proinflammatory phenotype is tightly linked to the progression of obesity-related disorders. An early study on ATDEVs already showed that these EVs were able to drive AT-resident macrophages into a proinflammatory phenotype secreting interleukin-6 (IL-6) and tumor necrosis factor (TNF-α) in a toll-like-receptor 4-dependent manner in macrophages. In this way, ATDEVs act thus as a mode of communication between the AT and the macrophages, which downstream leads to insulin resistance and a ADTEVs-mediated inflammation response. More specifically, toll-interleukin-1 receptor domain-containing adaptor protein inducing interferon-β (TRIF), used as an adaptor protein for the toll-like-receptor 4 signaling pathway, inhibited induction of IL-6 and TNF-α. Moreover, the specific TRIF pathway is known to activate the nuclear factor-kappa B (NF-κB) pathway, a prototypical proinflammatory signaling pathway inducing a variety of proinflammatory genes, leading to the secretion of cytokines and chemokines involved in the immune response. Interestingly, NF-κB signaling is associated with BBB alterations, comprising tight junction and adhesion protein impairments (Deng et al., 2009). Additionally, murine ATDEVs were found to induce macrophage proinflammatory polarization through modulation of the NF-κB pathway. They found that ATDEVs cause intracellular accumulation of lipids in the macrophages through altered protein expression of cholesterol efflux transporters leading to the formation of macrophage foam cells (Xie et al., 2018). Finally, elevated levels of micro-RNA-34a (miR-34a) were found in ATDEVs from obese mice, which transferred miR-34a to AT-resident macrophages, causing a switch from the anti-inflammatory state into the proinflammatory phenotype in AT-resident macrophages, leading to inflammatory cytokine secretion, local AT fibrosis and downstream AT dysfunction including insulin resistance and decreased adiponectin secretion (Pan et al., 2019). ATDEVs thus act as a mode of communication between the AT and the macrophages, which leads to downstream insulin resistance and a ADTEVs-mediated inflammation response.

Together, chronic inflammation, altered adipokine secretion, lipotoxicity, and oxidative stress contribute to the pathogenesis of AT dysfunction, thereby significantly increasing the risk for CVD. Over the past couple of years, it has become evident that ATDEVs play a central role in these pathways. For more information and a broad overview of ATDEVs involved in AT dysfunction, we refer to this review (Connolly et al., 2021). Of interest, the pathways involved in AT dysfunction are directly associated with EC dysfunction, which plays a central role in the development of CVD. In the next paragraph, we will provide a brief overview of ATDEV-mediated EC dysfunction mechanisms, and extrapolate this to the BBB, as an integral part of the neurovascular system.

**Mechanisms of adipose tissue–derived extracellular vesicles in vascular inflammation and dysfunction:** The association between AT, ATDEVs and the increased risk of CVD are well established, yet the underlying mechanisms are less well studied. Vascular inflammation is a central driver in the pathogenesis and progression of CVD, with chronic inflammatory processes directly contributing to EC dysfunction and vascular instability. Persistent inflammation within the vascular endothelium enhances permeability, promotes leukocyte recruitment, and activates platelets, fostering conditions like atherosclerosis and hypertension. This inflammation is further exacerbated by oxidative stress, which not only perpetuates endothelial damage but also fuels a self-reinforcing cycle of inflammatory cytokine and adhesion molecule production. Key inflammatory pathways, such as the NF-κB/IL-6 signaling axis, play a crucial role in this process, with immune cells like macrophages actively participating in the formation of atherosclerotic plaques and contributing to plaque instability. In a study by Wadey et al. (2019), researchers found an upregulation of VCAM-1 on ECs induced by ATDEVs. This effect was mediated by EV-derived TNF-α and resulted in an increased attachment of leukocytes to ECs. The main mode of action through which ATDEVs act on ECs is through the function of specific miRNAs in their cargo. For instance, ATDEVs from perivascular adipocytes have been shown to mediate vascular remodeling by contractile gene suppression of vascular smooth muscle cells, switching them into a more synthetic phenotype. Here, miR-221 mediates observed impairments by suppression of PGC-1ɑ, validated as a regulator of EC dysfunction and vascular smooth muscle cell proliferation through mitochondrial dysfunction. Perivascular AT, specific AT surrounding blood vessels is considered to have both a metabolic function as well as supporting the vascular tone (Li et al., 2019). Additionally, miR-27b packed in visceral ATDEVs induces endothelial inflammation. Here, the NF-κB pathway is activated, highlighting the importance of this signaling pathway in ATDEVs-mediated inflammation. Moreover, the expression levels of proinflammatory genes, including VCAM-1, interleukin-1β, and IL-6, were upregulated in ECs through ATDEVs. In this study, the peroxisome proliferation-activated receptor is the proposed target of miR-27b as peroxisome proliferation-activated receptor levels were decreased in the ATDEVs-treated ECs, and overexpression of peroxisome proliferation-activated receptor counteracted the mir-27b induced inflammatory response (Tang et al., 2023). Finally, a noteworthy point to mention is the uncovering of the adenosine A2A receptor (Adora2a) and its recognition of its pivotal role in the modulation of neurovascular and neuroinflammatory responses. Activation of Adora2a receptors in an obese mouse model has been associated with enhanced inflammatory signaling, leading to the disruption of the BBB (Yamamoto et al., 2019). The Adora2a receptor is not yet directly established in ATDEVs; however, they have been uncovered in EVs in the plasma of coronary artery disease patients, highlighting the need for future studies.

**Figure 1:**
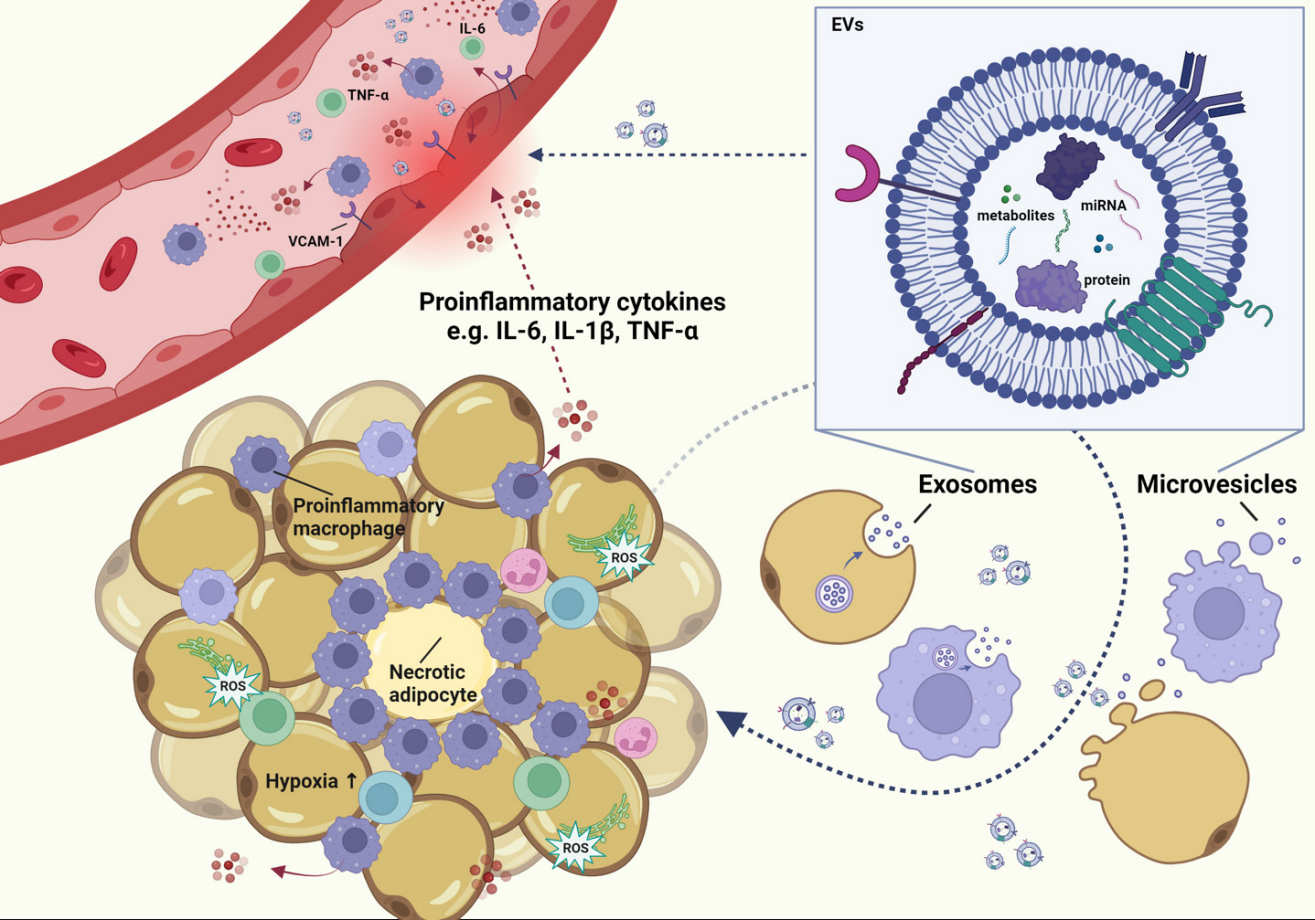
Pathophysiological changes in the obese adipose tissue and vascular endothelial cells through the action of obesity-associated ATDEVs. ATDEVs, secreted by adipocytes, immune cells, and, in small amounts by others, contain specific cargo including proteins, bioactive metabolites, and miRNA that affect the adipose tissue and endothelial cells leading to adipose tissue dysfunction and vascular inflammation. Endoplasmic reticulum stress is triggered by ATDEVs, causing the formation of reactive oxygen species leading to adipose tissue dysfunction and endothelial damage. Anti-inflammatory macrophages are switched to a proinflammatory phenotype by ATDEVs, secreting proinflammatory cytokines (e.g., IL-6, IL-1β, and TNF-ɑ), which aggravate vascular inflammation. Created with BioRender.com. ATDEVs: Adipose tissue-derived extracellular vesicles; EVs: extracellular vesicles; IL: interleukin-1; miRNA: micro ribonucleic acid; TNF-ɑ; tumor necrosis factor-alpha; VCAM-1: vascular cell adhesion molecule-1.

**Summary:** Since vascular inflammation significantly contributes to the onset and progression of neurodegenerative disorders, including MS, we postulate that ATDEV-mediated vascular impairment could be extrapolated to the BBB. Indeed, obesity, a well-known risk factor for EC dysfunction, has been shown to induce BBB disruption and promote neuroinflammation by increasing its permeability. Recent research highlights the role of ATDEVs in obesity-related vascular inflammation, which contributes to CVD and, by extrapolation, could potentially impair the BBB. Although obesity-related ATDEVs are known to be involved in vascular inflammation (**Figure 1**), and obesity being a pivotal risk factor for the development of MS, the direct link between obesity-related ATDEVs and impaired BBB integrity has not been established. Of importance, clinical studies linking ATDEV to neurodegeneration have not been performed yet. The goal of our perspective was to shed light on key mechanisms involved in ATDEV-mediated vascular inflammation and EC dysfunction, which could potentially be involved in BBB impairment and inflammation. Future studies should aim to establish causality between ATDEVs and neurovascular dysfunction where they focus on the therapeutic potential of pathways and biomolecules involved in the AT-brain axis. This will not only have a huge impact on the scientific community, but also on patients. With this perspective, we have opened a new window for future research in the field of the complex obese adipose tissue-brain crosstalk.

*We would like to thank Kenneth Verboven (Hasselt University, Belgium), Kristiaan Wouters (Maastricht University, The Netherlands), Lisa Schütz (Hasselt University, Belgium and Maastricht University, The Netherlands), and Lisa Mennens (Hasselt University, Belgium and Maastricht University, The Netherlands) for fruitful discussions*.

*This work was supported by FWO (Fonds voor Wetenschappelijk Onderzoek), grant number G07562NFWO (to BB)*.
